# Blood pressure long term regulation: A neural network model of the set point development

**DOI:** 10.1186/1475-925X-10-54

**Published:** 2011-06-21

**Authors:** B Silvano Zanutto, Bruno Cernuschi Frías, Max E Valentinuzzi

**Affiliations:** 1 Instituto de Ingeniería Biomédica (IIBM), Facultad de Ingeniería (FI), Universidad de Buenos Aires (UBA), Paseo Colón 850, (C1063ACV) Ciudad de Buenos Aires, Argentina; 2 Instituto de Biología y Medicina Experimental (IBYME-CONICET), Consejo Nacional de Investigaciones Científicas y Técnicas (CONICET), Vuelta de Obligado 2490, (C1428ADN) Ciudad de Buenos Aires, Argentina; 3 Instituto Argentino de Matemática (IAM-CONICET), Saavedra 15-Piso 3, (1083) Ciudad de Buenos Aires, Argentina; 4 Laboratorio de Investigación en Procesamiento de Señales e Imágenes y Redes Neuronales, Facultad de Ingeniería (FI), Universidad de Buenos Aires (UBA), Paseo Colón 850, (C1063ACV) Ciudad de Buenos Aires, Argentina

## Abstract

**Background:**

The notion of the nucleus tractus solitarius (NTS) as a comparator evaluating the error signal between its rostral neural structures (RNS) and the cardiovascular receptor afferents into it has been recently presented. From this perspective, stress can cause hypertension via set point changes, so offering an answer to an old question. Even though the local blood flow to tissues is influenced by circulating vasoactive hormones and also by local factors, there is yet significant sympathetic control. It is well established that the state of maturation of sympathetic innervation of blood vessels at birth varies across animal species and it takes place mostly during the postnatal period. During ontogeny, chemoreceptors are functional; they discharge when the partial pressures of oxygen and carbon dioxide in the arterial blood are not normal.

**Methods:**

The model is a simple biological plausible adaptative neural network to simulate the development of the sympathetic nervous control. It is hypothesized that during ontogeny, from the RNS afferents to the NTS, the optimal level of each sympathetic efferent discharge is learned through the chemoreceptors' feedback. Its mean discharge leads to normal oxygen and carbon dioxide levels in each tissue. Thus, the sympathetic efferent discharge sets at the optimal level if, despite maximal drift, the local blood flow is compensated for by autoregulation. Such optimal level produces minimum chemoreceptor output, which must be maintained by the nervous system. Since blood flow is controlled by arterial blood pressure, the long-term mean level is stabilized to regulate oxygen and carbon dioxide levels. After development, the cardiopulmonary reflexes play an important role in controlling efferent sympathetic nerve activity to the kidneys and modulating sodium and water excretion.

**Results:**

Starting from fixed RNS afferents to the NTS and random synaptic weight values, the sympathetic efferents converged to the optimal values. When learning was completed, the output from the chemoreceptors became zero because the sympathetic efferents led to normal partial pressures of oxygen and carbon dioxide.

**Conclusions:**

We introduce here a simple simulating computational theory to study, from a neurophysiologic point of view, the sympathetic development of cardiovascular regulation due to feedback signals sent off by cardiovascular receptors. The model simulates, too, how the NTS, as emergent property, acts as a comparator and how its rostral afferents behave as set point.

## Introduction

In a previous review paper, we collected sufficient evidence to advance the notion of the nucleus tractus solitarius (NTS) as a comparator evaluating the error signal between its rostral neural structures (RNS) and the cardiovascular receptor afferents into it [1]. Mean arterial long-term blood pressure (MAP) is regulated by the feedback of chemo and cardiopulmonary receptors, and the baroreflex would stabilize the short term pressure value to the prevailing carotid MAP; besides, the discharge rates of RNS projections to the NTS would function as the set point of the closed and open loops of cardiovascular control. Even with a set point, since the feedback open loop gain is very low [2], the MAP can be modified varying such gain, so partially explaining why there are different ways to control hypertension. From this perspective, Guyton's question posed in 1991 [3] (*how can stress cause hypertension?*), when he noted *that many prominent researchers believe that much, if not most hypertension in human beings is initiated by nervous stress*, may find the following answer: If the set point changes.

Even though the local blood flow to tissues is influenced by the level of circulating vasoactive hormones and also by local factors, including metabolites and endothelial substances, there is yet significant sympathetic control, as well expressed by Hilton and Spyer, in 1980, and also by Shepherd, in 1983 [4,5] when the latter noted that "*the role of the sympathetic nerves may be to modulate the local dilator mechanism to maintain the most economical ratio of blood flow to oxygen extraction*". Kidneys, too, are under sympathetic control; they regulate the renal blood flow, the glomerular filtration rate, the reabsorption of water, sodium and other ions, and the release of renin, prostaglandins, and other vasoactive substances, as stated by DiBona, in 1982 [6]. It is well established that the state of maturation of sympathetic innervation of blood vessels at birth varies across animal species and it takes place mostly during the postnatal period, reported Bevan *et al*, in 1980 [7].

During ontogeny, chemoreceptors are functional; they discharge when the partial pressure of oxygen and carbon dioxide in the arterial blood are not normal, i.e., different from the average 95 and 40 mmHg, respectively, reported Itskovits and Rudolph, in 1987 [8], and Boekkooi *et al*, in 1992 [9]. These receptors are involved not only in the nervous control, but also in the renin mechanisms [10]. The baroreflex is also functional and the cardiovascular responses to perturbations of the feedback loop (such as electrical stimulation) are similar to those in adult animals, which was demonstrated by Shinebourne *et al *back in 1972 [11]. However, the baroreflex is not involved in the long-term regulation; a few authors, though, propose to revise such almost traditional assertion, as further discussed below. Cardiopulmonary reflex is impaired early in life and increases with maturation, but there is evidence that central integration of cardiopulmonary vagal afferent input is fully functional in the term fetal and newborn sheep [12]. During this period, the role of the humoral and autoregulatory mechanisms is particularly important because the nervous control is not fully developed, a fact well addressed by Geis *et al *[13], Dworkin [14] and Tucker and Torres [15], the latter in 1992. Some researchers postulate that plasticity during ontogeny is influenced by individual environmental interactions, as for example Cohen and Randal [16]. Besides, the developing regulatory neural mechanisms adapt to environmental and behavioral conditions, documented by Friedman *et al *[17], Dworkin and Miller [18] and later on, in 1977, by the same Dworkin [19].

Mannard and Polosa, in 1973 [20], found that the sympathetic efferent system in animals has a characteristic interval histogram with a mean discharge rate. In the model presented here, the development of the nervous control of each sympathetic efferent is shaped in such a way that its mean discharge rate leads to normal oxygen and carbon dioxide levels in each tissue. Thus, and it should be stressed, the sympathetic efferent discharge sets at the optimal level if, despite maximal drift, the local blood flow is compensated for by autoregulation. Such sympathetic efferent optimal level produces minimum chemoreceptor discharge and this is what the nervous system has to maintain. During ontogeny, the case may be that chemoreceptors discharge even when some tissues are well irrigated because some others are not. Nevertheless, if the system evolves so that the sympathetic efferent to each tissue under control has a discharge rate near the optimal level, fewer tissues will be poorly irrigated. When the role of the cardiopulmonary set is well understood, it could easily be included in the model because the latter converge into the same pool of central neurons, as the arterial receptors act in a similar way, according to Spyer in his 1981 and 1990 reports [21,22]. After development, the cardiopulmonary reflexes play an important role in controlling efferent sympathetic nerve activity to the kidneys and modulating sodium and water excretion, as shown by Kopp *et al*, in 1991, and by Peterson *et al*, in 1992, [23,24].

Herein, we introduce a simple neural network model to study from a neurophysiologic point of view how the NTS has the *emergent property *of a comparator and how its RNS afferent pathway signals act as the set point.

## Materials and methods

### Overall description

**Figure 1:**
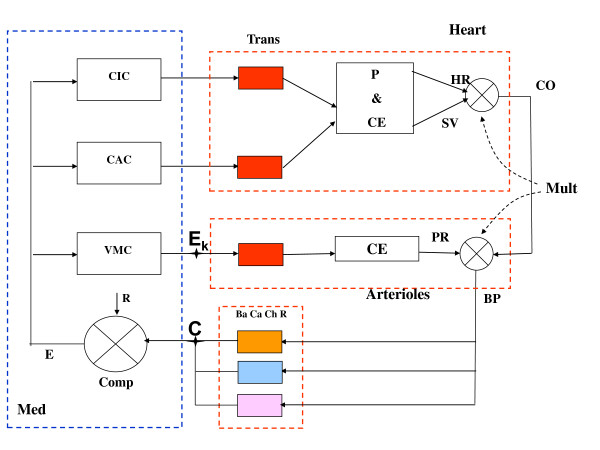
**Baro-Cardiopulmonary-Chemoreceptor Negative Cardiovascular Feedback Loop**. Med, medulla oblongata; CIC, CAC, VMC, cardioinhibitory, cardioaccelerator and vasomotor centers, respectively; Comp, comparator; neural reference R; error signal E after the difference against the outflow from the baro, cardiopulmonary and chemoreceptors (Ba, Ca ChR), respectively. BP, blood pressure = PRxCO; Mult, multipliers; PR, peripheral resistance; CO, cardiac output = HRxSV; HR, heart rate; SV, stroke volume. P, pacemaker; CE, myocardial contractile fibers; Trans, postulated transducers from neural section to CV side. Point C, output from the baro, cardiopulmonary and chemoreceptors; Point E_k_, output from the vasomotor center VMC, in the medulla. Reproduced after Ref (1).

**Figure 2:**
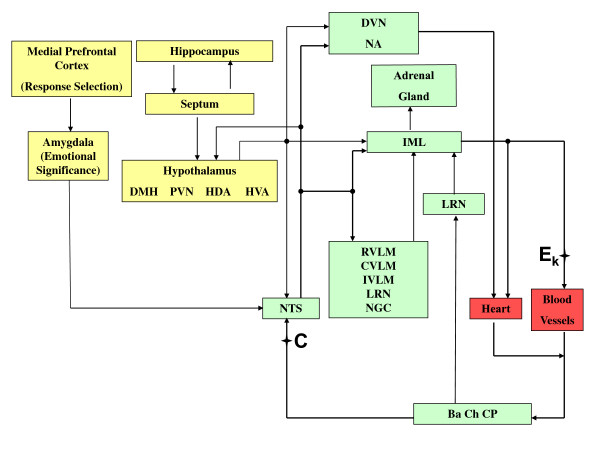
**Cardiovascular System Nervous Control**. The NTS receives afferents from its rostral nervous structures and send efferents to pre-sympathetic and pre-parasympathetic neurons. The same points C and E_k _link, respectively, to NTS (the comparator) and to the blood vessels.

**Figure 3:**
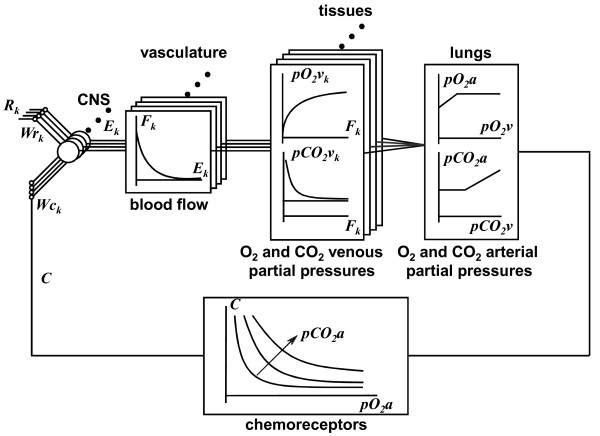
**Neural network model of the simpathetic regulation**. For each tissue (*k*), the sympathetic efferent discharge (*E_k_*), the blood flow (*F_k_*), the venous partial pressure of oxygen and carbon dioxide (*pO_2_v_k_*) and (*pCO_2_v_k_*), and the arterial partial pressure after the lung gases diffuse (*pO_2_a*) and (*pCO_2_a*) are shown. Finally chemoreceptors discharge (*C*) is depicted. *R_k _*represents the rostral neural nuclei inputs from the NTS, and *Wr_k _*and *Wc_k _*represent the synaptic weights.

This is a computer iterative model based on information collected from the literature at large and from the authors' own experimental data. To start with, let us refer to Figure 1, reproduced from Zanutto *et al *[1], where the output from the baro, cardiopulmonary and chemoreceptors (point C) is one of the inputs to the comparator and the output from the vasomotor center VMC, in the medulla (point E_k_), goes to the neuromechanical transducers that control the arteriolar contractile elements CE. If now, instead, we refer to Figure 2, also reproduced from Reference [1], those same points link, respectively, to the nucleus tractus solitarius NTS (the comparator) and to the blood vessels. Figure 3 is a functional diagram describing blood gases partial pressures, as a "tiny devil" imaginary observer moves from tissues to lungs to the chemoreceptors to the manifold inputs (point C) at the comparator level of the central nervous system CNS. Obviously, branching off at point C is manifold because of the huge number of nerve fibers with many outputs *E_k _*from the comparator elements after subtraction from the same number of references *R_k _*(which, in the end, conform the set point). Those signals *E_k _*(nerve action potentials at a given rate) act upon the vasculature smooth muscles to control the vessel lumens, which in turn, would let a higher or lower blood flow *F_k_*; hence, the relationship is inverse, i.e., the higher the neural discharge, the lower the blood flow through that particular arteriole to tissue *k*, where *k *is a running integer (1 <*k *< N). The vasculature supplies blood to the tissues where, as the blood flow increases, the partial pressures of oxygen and carbon dioxide, respectively, go up and down (Figure 3, middle part). Thereafter, blood returns to the pulmonary circulation to replenish its oxygen load and discharge the carbon dioxide overload. For oxygen, arterial partial pressure increases as its input venous counterpart goes up, however, beyond a given value of the latter, the former tends to a plateau because red cells reach saturation; for carbon dioxide, instead, the situation is different producing a concave relationship, that is, CO_2 _stays rather constant while venous partial pressure of CO_2 _gets higher until it starts to steadily grow after a given threshold. Clearly, the input partial pressures depend on each particular situation, say, normal resting condition, mild or heavy exercise, or perhaps a respiratory or metabolic pathology. Their arterial levels, after the pulmonary exchange takes place, are detected by the chemoreceptors (represented in the lower block parametric relationship) from where we get back to point C, as the weighted input to the different comparator neural cells.

### An adaptive model of the circulatory system nervous control

Some RNS links to the NTS are involved in learning as well as other nervous structures of the medulla and the mesencephalon [25,26]. However, it is not well known which nervous structures are involved. Anyhow, we are interested in simulating the plastic effect in a very simple and biologically plausible manner. In the simulation, we consider that the information fedback by chemoreceptors controls the plastic adaptive process in such a way that the synaptic changes will produce the optimal level of sympathetic efferent discharge. This process was simulated with a neural network, with one node to control each tissue bed or organ (to consider the general case, but one node could control blood flow to several tissues) and the plastic changes would take place in one synapse for each node. We assume that if there are saturation effects, they do not affect the plasticity during development. Because of that, it is also supposed that the error signal (between the RNS projections to the NTS and the feedback inputs) controlling each tissular bed would cause similar proportional effect over each sympathetic efferent.

### Computer simulation

The model is represented in Figure 3, where only those structures involved in the plastic process are considered for a given time *t*. There are *N *nodes and each node *k *has two inputs, one from the chemoreceptors and another from the RNS to the NTS of the CNS. The node output *E_k _*represents the mean discharge rate of the sympathetic efferent that controls blood flow to that tissue *k*. During development, the system produces an *E_k _*to cause minimum chemoreceptor outflow. From the *E_k _*to a given tissue, its blood flow *F_k _*is simulated by a hyperbola (Figure 3, left-hand block; see also Celander, [27]), that is,(1)

where *E_k _*takes values between 0.05 and 1; the numerical value 0.05 is an arbitrary small constant and 1 bounds the curve to a non-zero level. Under normal metabolism, the describing curves of oxygen and carbon dioxide partial pressures at the capillary venous end (including the interstitial fluid), both as functions of blood flow, are assumed to be the same for all beds (typical of any well-behaved exchanger). Hence, gas partial pressures within each tissue are simulated by an exponential and by a hyperbola, respectively (Figure 3, middle block, top and bottom curves; see [28], Guyton, 1986), so that the following equations can be written down,

where *pO_2_v_k _*and *pCO_2_v_k _*are both measured in mmHg. These represent the average values, but in extreme cases as in muscles under vigorous exercise, the *pO_2_v_k _*could drop as low as 3 mmHg whereas *pCO_2_v_k _*could increase up to 90 mmHg [29]. Besides, it is assumed that the central venous partial pressures result from the Gaussian random additional mixture of the venous blood pressure in all tissues. Thus, the equations used were as follows,

each, respectively, describing the venous oxygen and carbon dioxide partial pressures, where *n_k_*'s are Gaussian random numbers between 0 and 1, with a mean value of 1, and a standard deviation of 0.5 (underlining that only the left half of the curve is considered). The arterial gas partial pressures coming out from the lungs are calculated as functions of the central venous partial pressures. As blood flows along the pulmonary capillaries, it receives oxygen from the alveoli and delivers carbon dioxide to them. Thus, concentration gradients for these two gases develop along the pulmonary capillaries, increasing for oxygen and decreasing for carbon dioxide. The perfusion of oxygen *O_2 _*follows a potential function while carbon dioxide *CO_2 _*does it as a decremental exponential function (see Milhorn and Pulley, Ref 30).

If the partial pressures of oxygen *pO_2 _*and carbon dioxide *pCO_2 _*in the alveoli are 104 mmHg and 40 mmHg, respectively, and the venous blood pressures are *pO_2_v *= 40 mmHg and *pCO_2_v *= 45 mmHg, then the arterial blood partials would be normal, that is, 95 mmHg for oxygen *pO_2_a*, and 40 mmHg for carbon dioxide *pCO_2_a*. This phenomenon occurs in approximately one third of the time available to diffuse [30]. Therefore, even for values of *pO_2_v *lower and *pCO_2_v *higher than those mentioned above, if there is enough time to diffuse, the arterial partial pressures would be normal. With shorter diffusion times or if *pO_2_v *is lower than a threshold and *pCO_2_v *is higher than another threshold, gas diffusion would not be sufficient and the exchange becomes impaired. Since in the present model these phenomena occur within a feedback loop, knowing their exact mathematical form loses significance. Herein, when *pO_2_v *is lower than 39.55 mmHg and *pCO_2_v *is higher than 45.2 mmHg, gas diffusion in the alveoli is taken as abnormal. In this case, the arterial partial pressures vary from the optimal levels following a linear law, that is,(6)

and(7)

Chemoreceptors increase their discharge exponentially if the *O_2 _*concentration decreases or *CO_2 _*concentration increases from the normal levels [31]. This function is formalized as,(8)

where(9)

and(10)

The output from each network node *k *is calculated as the difference between its two inputs [32] or, in mathematical form,(11)

where *R_k _*(which is a random number ranging from 0.1 to 0.9) is the discharge rate of a RNS afferent, *C *represents the chemoreceptor discharge rate, *E_k _*stands for the axon discharge rate, *Wr_k _*is the synaptic weight of the RNS input, and *Wc_k _*is the chemoreceptor synaptic weight. The function *G *is chosen as a sigmoid, that is,(12)

The values of *Wc_k _*are equal to 1 and those of *Wr_k _*are calculated based on Hebb's law [33], in turn accepting also experimental evidence, as that given by Brown *et al *[34]. The usual formalization of this law assumes that the synaptic weight increases if there is activity both at the output and input of the neuron simultaneously; however, the latter is not realistic because it only allows unidirectional changes. Kohonen, back in 1982 and 1988 [35,36], modified this formula adding a negative term proportional to the synaptic weight multiplied by the output value of the node and assuming that *there exists a competition on synaptic resources within the cell*. He also said that it resembles the teaching rule of the *Perceptron *[37] except that the corrections are always in the same direction of the input values. Here, for each neuron *k*, the synaptic weight has to maintain its value if there is no chemoreceptor discharge. If the gases partial pressures are not correct, *C *is greater than zero, and the synaptic weight has to be diminished. Because of that, the coefficient *β_t _*in Equation (13) below is multiplied by (1 + *C*). In this way, the synaptic weight in each iteration *t *is calculated as,(13)

Where  are the values of *Wr*_*k *_to be computed in the next iteration (*t+1*) and  are the values of *Wr*_*k *_in the actual iteration (*t*, as used in equation 11). Besides, *E*_*k *_and *C *stand for values of the current iteration. To show the effect of the synaptic weight in the plasticity process, the values of *α_t _*and *β_t _*are considered as constant. Besides, to simulate learning in a critical period, these values have to decrease with time *t*; say, they could follow a hyperbolic function. In both cases, there are no differences in the simulated values of the partial pressures of gases and the sympathetic and chemoreceptor discharge rates stabilize in the same way.

## Results

**Figure 4:**
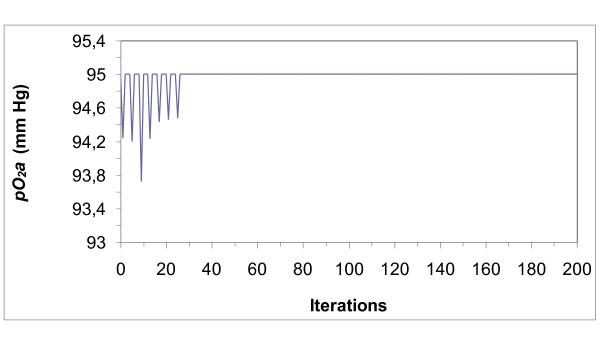
**Arterial partial pressure of oxygen (*pO*_*2*_*a*)**. Partial pressure evolves with fluctuations until the optimal value is achieved. The figure shows the dynamics of the first 200 iterations until the system adapts (when the discharge when the frequency of chemo receptors fall to zero). For each animal maturation takes place in a given time T, since 1,000 iterations were run, the unit is T/1,000. This comment applies to the rest of the figures.

**Figure 5:**
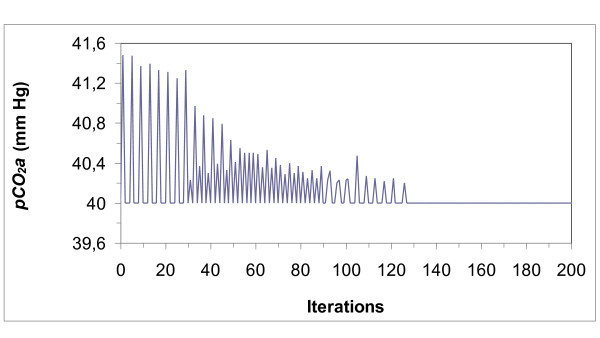
**Arterial partial pressure of carbon dioxide (*pCO*_*2*_*a*)**. Partial pressure, before arriving at its maximum value, evolves like chemoreceptor discharge. Pressure converges to the optimal value.

**Figure 6:**
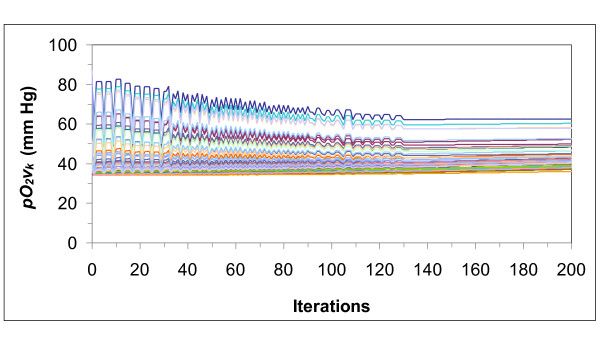
**Venous partial pressure of oxygen (*pO*_*2*_*v*_*k*_) of each tissue (*k*)**. The partial pressures start from random values and converge to the optimal values that provoke minimum chemoreceptor discharge.

**Figure 7:**
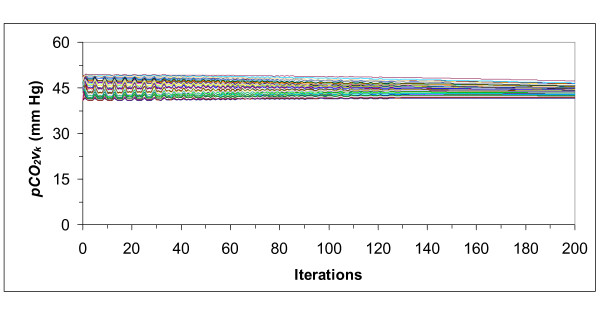
**Venous partial pressure of carbon dioxide (*pCO*_*2*_*v*_k_) of each tissue (*k*)**. Partial pressures start from random values and converge to the optimal values that provoke minimum chemoreceptor discharge.

**Figure 8:**
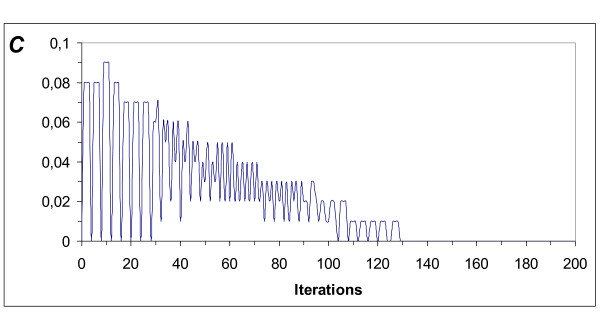
**Chemoreceptor discharge rate (*C*)**. When arterial partial pressures drift from the normal values, the chemoreceptors discharge, and do so until equilibrium is reestablished. The chemoreceptor discharge rate reaches a maximum value, then slowly and with fluctuations decays to zero when learning is finished. Measured in arbitrary values between 0 and 1.

**Figure 9:**
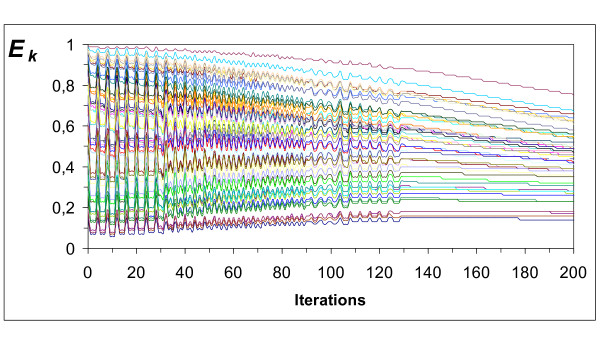
**Sympathetic efferent discharge rate (*E*_*k*_) of each tissue (*k*)**. The discharge rate values evolve so that, after some fluctuations, all converge. Measured in arbitrary values between 0 and 1.

The model only simulates the sympathetic development process; it does not include any other type of regulation [1]. The evolution of cardiovascular variables due to plasticity, arterial and venous blood partial pressures (Figures 4, 5, 6, &7) and chemoreceptor discharge rate (Figure 8) were simulated for *N *= 50 tissues by iterations, from 1 to 1000. Learning was accepted as completed once the partial pressures of gases reached optimal asymptotic values with no chemoreceptor discharge. To simulate the delay of closed loop, C was computed as the mean value of the last 3 iterations. The constant values to compute the synaptic weight were *α_t _*= 0.001 and *β_t _*= 0.005. Starting from random synaptic weight values for *Wr_k _*between 0 and 1, the node outputs *E_k _*converged to the optimal values. In the successive iterations, the values of *Wr_k _*and the node outputs *E_k _*converged always to fixed values. In the first 130 iterations, *E_k _*converged in an oscillatory way (Figure 9), followed by the chemoreceptor discharge rate (Figure 8). Convergence continued without oscillations up to the end near the 1000th iteration. When learning was completed, the output from the chemoreceptors became zero because *E_k _*leads to normal partial pressures of oxygen (Figures 4 and 6) and carbon dioxide (Figures 5 and 7). These results are independent of the number of tissues.

To simulate learning in a critical period, the values of *α_t _*and *β_t _*must diminish with time *t*. For example, they could follow a hyperbolic function. In the simulation, there are no differences in the evolution of the partial pressures of gas values. Furthermore, the sympathetic and chemoreceptor discharge rates stabilize in the same way. In this case, *C *can be removed from the learning rule (Equation 13) without any important change in synaptic weights. The only significant change appears when the neural network outputs have very small oscillations, depending on the values of *α_t _*and *β_t_.*

## Discussion and conclusions

Physiological experiments show that denervation of all the cardiovascular receptors (baro, chemo and cardiopulmonary) leads to sustained hypertension. Based on this fact, in a previous work we concluded that mean long-term blood pressure is regulated by the nervous system [1]. In that paper, both from a neurophysiologic and from a control theory point of view, it was shown that the Nucleus Tractus Solitarius (NTS) acts as a comparator, evaluating the error signal between its RNS afferents and the cardiovascular receptors. It is so that these rostral afferents to the NTS would function as the set point of cardiovascular regulation. Furthermore, it has been well established that the maturation state of blood vessels sympathetic innervation at birth varies across animal species, taking place mostly during the postnatal period [7]. On these bases, we are introducing a simple neural network model to study from a neurophysiological point of view how the NTS has the emergent property of a comparator and how its RNS afferents act as the set point. In the model, during ontogeny of the cardiovascular regulation, the nervous control adapts from the information fedback into the NTS by chemoreceptors (and perhaps also by cardiopulmonary receptors) activity. Specifically, from fixed values (chosen at random) representing the RNS afferents, the mean discharge rates of the sympathetic efferents are adjusted through the chemoreceptor feedback varying the synaptic weights. Their discharge rates have to maintain tissue blood flows so that the autoregulatory mechanisms can regulate the partial pressures of oxygen and carbon dioxide to normal values (pO_2 _= 95 mmHg and pCO_2 _= 40 mmHg, for arteries, and pO_2_v = 40 mmHg and pCO_2_v = 45 mmHg, for veins).

Similar results could have been obtained assuming different plasticity functions. We chose a simple function to test our hypothesis; however, when more neurobiological data are available, a better simulation could be obtained. During development, the renal curve would shift; this effect can be considered in the model by adding a constant to the curve of blood flow as a function of the sympathetic discharge. Thus, the system would adapt to any renal output curve change. The fact that the cardiovascular receptors adapt does not affect the model due to the feedback, since in this model it is sufficient that the NTS receives information about partial pressure changes. Nevertheless, this effect can also be easily included. In the model, the learning process could happen not only during ontogeny but also when the system readapts to a new condition (if this was the case, constants *α_t _*and *β_t _*would not have to diminish to zero).

In this model and during development, the partial pressures of O_2 _and CO_2 _are regulated only by the information provided by the chemoreceptors (and maybe by the cardiopulmonary ones) Even though this regulation is not as good as after ontogeny is completed, it is good enough to control the cardiovascular variables in the fetus and in the newborn. When ontogeny is finished, because of plastic changes in the sympathetic efferent discharge rates, mean arterial blood flows (which come up as an overall result) are regulated to keep the optimal partial pressures by the information provided via the chemoreceptors (and maybe by the cardiopulmonary ones).

In the cardiovascular system, blood flow is controlled by arterial blood pressure, and in this way the long-term mean blood pressure is stabilized to regulate oxygen and carbon dioxide levels. Thereafter, the baroreflex would stabilize the instantaneous pressure value to the prevailing carotid pressure (MAP). Baroreceptors appear as points of contention, too, that are unimportant in determining the long-term level of MAP. However, Terry Thrasher, cites and discusses studies in intact experimental animals suggesting that their resetting may not be as rapid or complete as previously thought [38]. Results, obtained using a new model of chronic baroreceptor unloading, indicate that the condition ends up in a sustained increase in MAP. These results suggest that the role of baroreceptors in the long term control of MAP needs to be revisited. If the baroreceptors have any role in the long-term regulation, they could easily be included in the model because the latter converge into the same pool of central neurons, as the arterial receptors act in a similar way. In this model, they would affect the dynamic of the feedback loop, but the cardiovascular variables would converge to the same values.

In the model, after development, the discharge rates of the RNS projections to the NTS functions as the set point of the open and closed loop of cardiovascular control. The same brainstem nuclei that project fibers to the sympathetic and the parasympathetic systems of the feedback loop are involved in a feedforward control [[1]; Figure 3]. The effect of these open loop projections can be simulated shifting the output values of the nodes proportionally to the *R_k _*values involved in this process. Since feedback is too slow to be involved in fast cardiovascular control reactions, the anticipatory feedforward action would play an important role in such response. In cardiovascular control there are modulation effects that would be easily simulated adding inhibitory and/or excitatory projections from the RNS of the feedback loop.

In our previous paper on this very subject [1], we stated that the long-term blood pressure regulation is controlled by the nervous system. Such view somewhat differs from Guyton's school, without ever disregarding the unquestionable involvement of diuresis and natriuresis. Even more, any pharmacological agent active in the latter mechanisms does it so because of the low loop gain. No doubt, there are still well-respected and versed supporters of the "kidney-centric" view, such as Montani and Van Vliet and Osborn *et al *[39]. However, in the light of overwhelming evidence for a major role of the sympathetic nervous system in long-term control of arterial pressure and the pathogenesis of hypertension, new theories for long-term control of arterial pressure may be necessary. Despite the prominence and general acceptance of the Guyton-Coleman model in the field of hypertension research, Osborn *et al *[40] argued that it overestimates the importance of renal control. Furthermore, they suggested that it is possible to construct alternative models in which sympathetic nervous system activity plays an important role in long-term control of arterial pressure. The latter two papers appeared in a single issue where both standings were amply discussed.

About the set point and comparator concepts, Dampney *et al *[41], in 2002, stated that the optimal level of arterial pressure is presumably determined by a balance between the need of an adequate perfusion pressure and by the fact that, as pressure increases, cardiac work and risk of damage to the heart and vessels also increases. Besides, they added that the level around which arterial pressure is regulated, the "set point", varies under different conditions. During dynamic exercise, arterial pressure is increased by approximately 15-20% for the benefit of a better blood flow to skeletal muscles. Non the less, there are rather older antecedents, such as contributions put forward by Zanutto *et al*, especially regarding the neural comparator [42-44]. In a similar direction (but without finding any mention to the comparator neural concept), Gregory Fink, states that the ultimate goal of cardiovascular regulatory mechanisms is the maintenance of tissue blood flows commensurate with metabolic requirements. Thus, elevated BP can contribute to optimizing tissue blood flows. Perhaps part of the rise in pressure is reflexly driven by a homeostatic mechanism to regulate tissue blood flows. In this way, the average long-term level of BP is an emergent property of a decentralized control system. Besides, these authors contend that the ability to generate a hypertensive phenotype increases the species lifespan [45,46].

There remain aspects still to be addressed that call for further research and open discussion, as for example, how the model could be tested and what predictions would be possible out of it. The main hypothesis of the model could be tested after finding the plastic synapses involved in development of the sympathetic cardiovascular regulation. It is that during that period, the plasticity is modulated by the feedback of the cardiovascular receptors in such a way that sympathetic afferents regulated the blood flow to optimal values.

Rounding out and in short: We introduce a simple computational theory to simulate, from a neurophysiologic point of view, the sympathetic development of cardiovascular regulation from the feedback of cardiovascular receptors. After development, the sympathetic discharge rates control arterial blood pressure to maintain tissue blood flows so that the autoregulatory mechanisms can adjust the partial pressures of oxygen and carbon dioxide to normal values.

## Symbols

*α_t_*: constant; β_t_: constant; *C*: chemoreceptor discharge rate; *E_k_*: axon discharge rate of neuron "*k*"; *F_k_*: blood flow of tissue "*k*"; *G*: sigmoid function; *N*: number of neurons; *pCO_2_a*: arterial carbon dioxide partial pressure; *pCO_2_v*: central venous carbon dioxide partial pressure; *pCO_2_v_k_*: carbon dioxide partial pressure at the venous end of capillaries in tissue "*k*; *pO_2_a*: arterial oxygen partial pressure; *pO_2_v*: central venous oxygen partial pressures; *pO_2_v_k_*: oxygen partial pressures at the venous end of capillaries in tissue "*k*"; *R_k_*: discharge rate of rostral neural structures afferent *"k*"; *S_k_*: output from node *"k"; t*: iteration counter; *Wc_k_*: chemoreceptor input *"k" *synaptic weight; *Wr_k_*: rostral neural structures input *"k" *synaptic weight; NTS: Nucleus of Tractus Solitarius; RNS: Rostral Neural Structures.

## Competing interests

The authors declare that they have no competing interests.

## Authors' contributions

BSZ formulated the hypotheses, developed the mathematical model, performed the simulation, interpreted the data and drafted the manuscript. BCF revised the hypotheses critically, supervised the simulations, interpreted the data and revised the manuscript. MEV participated in the interpretation of the data, revised the manuscript critically and participated of the final version of the manuscript. All authors read and approved the final manuscript.
